# LC-N2G: a local consistency approach for nutrigenomics data analysis

**DOI:** 10.1186/s12859-020-03861-3

**Published:** 2020-11-17

**Authors:** Xiangnan Xu, Samantha M. Solon-Biet, Alistair Senior, David Raubenheimer, Stephen J. Simpson, Luigi Fontana, Samuel Mueller, Jean Y. H. Yang

**Affiliations:** 1 School of Mathematics and Statistics, The University of Sydney, Sydney, NSW 2006 Australia; 2 Charles Perkins Centre, The University of Sydney, Sydney, NSW 2006 Australia; 3 School of Life and Environmental Sciences, The University of Sydney, Sydney, NSW 2006 Australia; 4 Sydney Medical School, The University of Sydney, Sydney, NSW 2006 Australia; 5 Department of Mathematics and Statistics, Macquarie University, Sydney, NSW 2109 Australia

**Keywords:** Nutrigenmoics, Local consistency, Nutrition, Gene expression

## Abstract

**Background:**

Nutrigenomics aims at understanding the interaction between nutrition and gene information. Due to the complex interactions of nutrients and genes, their relationship exhibits non-linearity. One of the most effective and efficient methods to explore their relationship is the nutritional geometry framework which fits a response surface for the gene expression over two prespecified nutrition variables. However, when the number of nutrients involved is large, it is challenging to find combinations of informative nutrients with respect to a certain gene and to test whether the relationship is stronger than chance. Methods for identifying informative combinations are essential to understanding the relationship between nutrients and genes.

**Results:**

We introduce Local Consistency Nutrition to Graphics (LC-N2G), a novel approach for ranking and identifying combinations of nutrients with gene expression. In LC-N2G, we first propose a model-free quantity called Local Consistency statistic to measure whether there is non-random relationship between combinations of nutrients and gene expression measurements based on (1) the similarity between samples in the nutrient space and (2) their difference in gene expression. Then combinations with small LC are selected and a permutation test is performed to evaluate their significance. Finally, the response surfaces are generated for the subset of significant relationships. Evaluation on simulated data and real data shows the LC-N2G can accurately find combinations that are correlated with gene expression.

**Conclusion:**

The LC-N2G is practically powerful for identifying the informative nutrition variables correlated with gene expression. Therefore, LC-N2G is important in the area of nutrigenomics for understanding the relationship between nutrition and gene expression information.

## Background

Nutrients are simple organic compounds involved in biochemical reactions that produce energy or are constituents of cellular biomass [1]. Nutrigenomics, the combination of nutrition and genomics research, which aims to shed light on and describe, characterize, and integrate the interactions between nutritional compounds and genome-wide gene expression [2], has been thriving after the completion of the human genome 15 years ago [3, 4]. This research led to a better understanding of the processes and mechanisms that define the relationships between nutrition, genetics, and physiology. Recently, some researchers use public databases further investigate how nutrients intake shape the interested gene expression [5, 6]. Nutrigenomics holds great promise to provide individualized plan to improve health (precision health) [7].

**Fig. 1:**
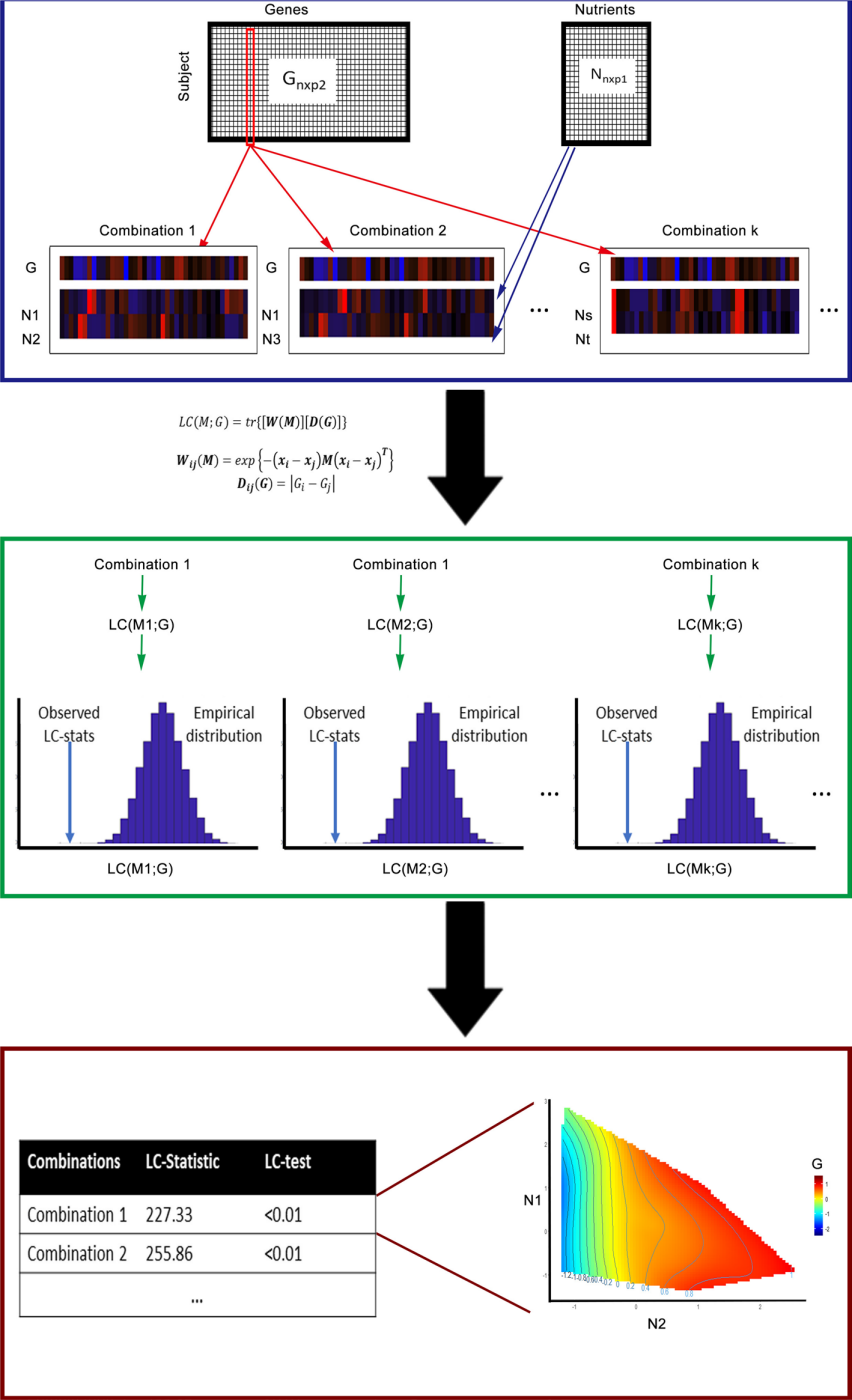
Overall workflow of LC-N2G. $G_{n \times p_2}$ and $N_{n \times p_1}$ represent input of matrix of gene and nutrition respectively. First step we calculate LC-Stat of combinations with a gene of interest to find combination of nutrients with small LC-Stat. Then a LC-Test is performed to evaluate the relationship between combination of nutrients with gene. Finally the NGF is performed for selected combination and genes

The relationship between the mechanisms of nutrients and genes is complex. For example, there are many potential non-linear interactions which are challenging for any bivariate measure of association such as Pearson and Spearman correlation [8]. One recent approach that addresses this challenge is the Nutritional Geometry Framework (NGF) proposed by Simpson and Raubenheimer [9, 10], where the nutritional requirements and their response are represented graphically in a pre-specified *k*-dimensional space. Each of these dimensions is a nutrient or some other dietary constituent. For example, the NGF shown in Fig. 4a is a two-dimensional colour graph and visualizes the relationship of two nutrients, Protein and Carbohydrate, on the expression level of a particular gene, FGF21. These two nutrients are represented on the x and y axis, respectively. The gene expression level is highlighted through a surface using a colour scale [9]. This framework makes available tools to interpret how nutrients and other dietary constituents, directly or through their interactions influence a given phenotype. The success of NGF has been demonstrated by much recent research [11–16]. However, this NGF framework requires manual selection of informative combinations of nutrients prior to visualization. This often requires deep domain knowledge and when the number of nutrients *p* is large, considering all pairwise combinations $$p(p-1)/2 = \left( {\begin{array}{c}p\\ 2\end{array}}\right)$$ becomes time consuming.

**Fig. 2:**
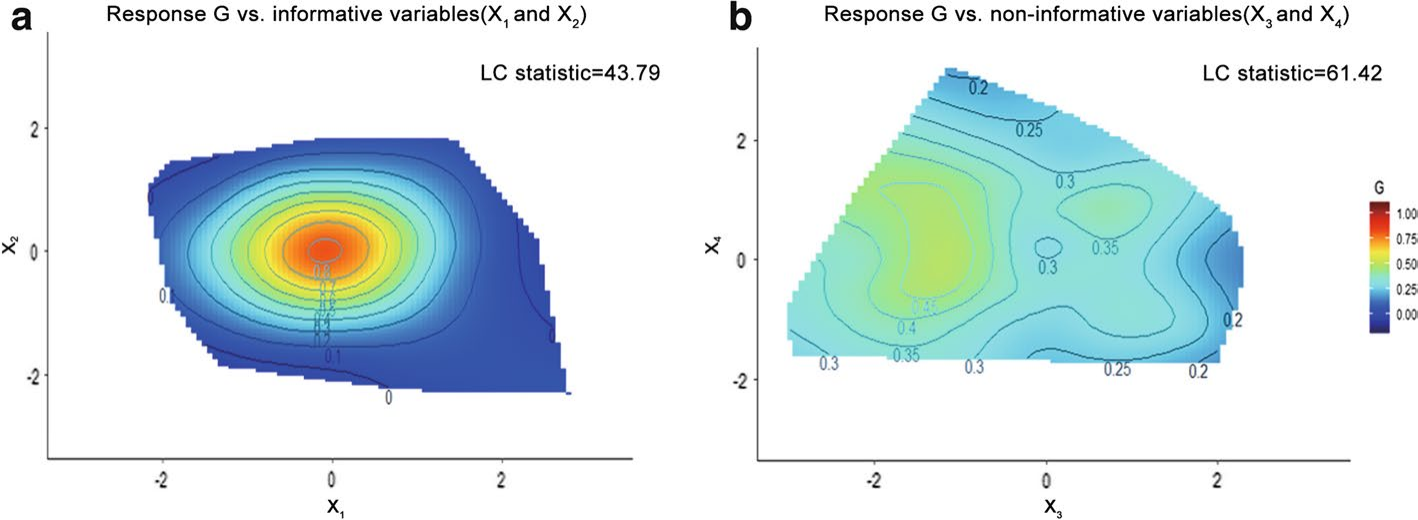
Illustration of LC-Opt and LC-Test. **a** and **b** are the NGF of a simulated data which **a** and **b** use the same response G while the covariate of **a** is informative and **b** is random. In this figure, thin plate spline are used to fit the curve

In this paper, we will address this challenge by proposing the **L**ocal **C**onsistency **N**utrition to **G**raphics (LC-N2G) approach to identify nutrition variables and use these to inform on changes in genes, that is achieving “nutrition to graphics”, visualising multivariate nutrition-gene relation. LC-N2G first constructs a Local Consistency statistic (LC-Stat) that measures the smoothness of a given gene expression surface relative to a set of axes that represents combinations of nutrients. Then optimization methods, in short LC-Opt, can be used for choosing combinations with smallest LC-Stat. Next a permutation based test, LC-Test, is used to evaluate whether the relationship between the gene expression and a combination of nutrients is significantly different from the relationship between a randomly permuted gene expression vector and this combination. Finally, NGF is used to visualize selected combinations with gene expression. We use both simulation and real data to validate our proposed LC-N2G method and the results show that it can correctly identify the relationship between gene and nutrients. The overall workflow is illustrated in Fig. 1.

## Results

### Local consistency statistics

Let matrix ${\varvec{X}}_{n\times p}$ denote the nutrition matrix where *n* is the number of rows (observations/samples), *p* the number of columns (nutrients/features), ${\varvec{x}}_i,i=1,\ldots ,n$, denote the rows of the nutrition matrix and ${\varvec{G}}_{n\times 1}$ denotes the response, i.e. one particular gene expression vector. We define LC-Stat as follows:

1 $$\begin{aligned} LC(M;G) = {\text {tr}}\{[({\varvec{W}}({\varvec{M}})][ {\varvec{D}}({\varvec{G}})]\}, \end{aligned}$$

where ${\text {tr}}$ denotes the trace of a matrix and the elements of the matrices ${\varvec{W}}({\varvec{M}})$ and ${\varvec{D}}({\varvec{G}})$ are given by

2 $$\begin{aligned} {\varvec{W}}_{ij}({\varvec{M}})&= \exp \{-({\varvec{x}}_i-{\varvec{x}}_j){\varvec{M}}({\varvec{x}}_i-{\varvec{x}}_j)^T \},\ \text {and} \end{aligned}$$

3 $$\begin{aligned} {\varvec{D}}_{ij}({\varvec{G}})&= \vert G_i - G_j \vert ,\quad (i,j=1,\ldots n), \end{aligned}$$

where ${\varvec{x}}_i$ and ${\varvec{x}}_j$ are the *i*th and *j*th row of ${\varvec{X}}_{n\times p}$, $G_i$ and $G_j$ are the *i*th and *j*th element of ${\varvec{G}}_{n\times 1}$, and ${\varvec{M}}$ is a diagonal matrix consisting of 1’s and 0’s on the diagonal only. For example if the first and the last nutrient is selected and *p* = 4 then

$$\begin{aligned} {\varvec{M}}=\begin{pmatrix} 1 &{} 0 &{} 0 &{} 0 \\ 0 &{} 0 &{} 0 &{} 0\\ 0 &{} 0 &{} 0 &{} 0 \\ 0 &{} 0 &{} 0 &{} 1 \\ \end{pmatrix}. \end{aligned}$$

Therefore, such a matrix ${\varvec{M}}$ represents an indicator of subspace. The LC-Stat is a function of both the indicator matrix ${\varvec{M}}$ and the given gene expression ${\varvec{G}}$. When ${\varvec{G}}$ is fixed, we can optimize ${\varvec{M}}$ to find the combination of nutrition variables with minimal LC-Stat (see Method LC-Opt). On the other hand, for a given combination of nutrition information, a permutation test can be performed to evaluate the significance of the relationship between this particular combination and ${\varvec{G}}$ (see Method LC-Test).

**Fig. 3:**
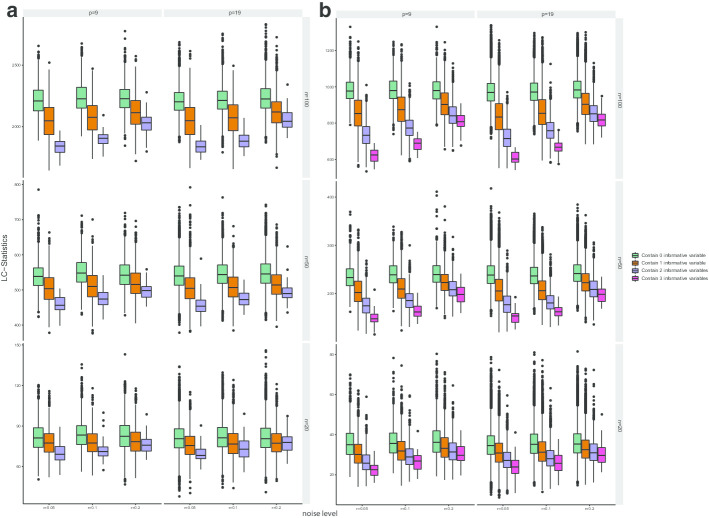
Simulation results for LC-Opt for identifying combinations. The combinations are divided into four groups according to the informative variables it included. **a** is box plot of LC statistics for different combination groups of Model 1. **b** is box plot of LC statistics for different combination groups of Model 3 with *k* = 3. In **a** and **b** total number of informative variables is 2 and 3 respectively

The basic idea of LC-Stat is that given a gene of interest with respect to certain nutrition variables, the response is smooth within a neighborhood in the nutrient space, i.e. for nearby observed points in the nutrient space, the response varies little. From the expression of LC-Stat and focusing on the term in Eq. (2), we can see that if two points are distant in the nutrient subspace selected by ${\varvec{M}}$, no matter their difference in response, its corresponding ${\varvec{W}}_{ij}$ term will contribute approximately zero to the total of LC-Stat. However, when two points are close, their difference in response will contribute with a substantial proportion to the value of the LC-Stat. Thus, for a given dataset, a smaller LC-Stat value means that data points have slowly changing response in their neighbourhood while a larger LC-Stat means that some data points have dramatically changing response in their neighbourhood.

**Fig. 4:**
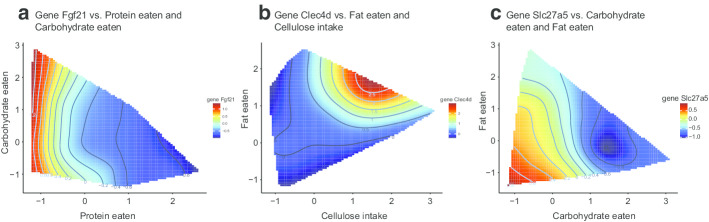
LC-N2G results for mouse nutrition study. **a**–**c** NGF of Fgf21, Slc27a5 and Clec4d with the informative combination identified by LC-N2G. Fgf21 is investigated in [15]. Slc27a5, Clec4d are hub genes by WGCNA

Figure 2 provides an illustration of how the LC statistic works. In this figure, a total of four normally distributed random variables, $X_1,X_2,X_3$, and $X_4$, are considered and the response, *G*, is generated by $G = \exp \{-(X_1^2+X_2^2) \}+0.1\epsilon$, where $\epsilon$ is a standard normally distributed noise term. Figure 2(a) shows the NGF of the response *G* with respect to the two informative variables $X_1$ and $X_2$, whereas Fig. 2b shows the same response *G* with respect to the two randomly generated non-informative variables $X_3$ and $X_4$. We can see clearly that Fig. 2a shows smoother transition across this 2-dimensional space and does not show a surface with high variability. Its corresponding LC statistic is 43.79. In contrast, multiple peaks and troughs can be seen when choosing random nutrition variables such as in Fig. 2b. In this situation also a much larger LC statistic of 61.42 is observed.

### Results on simulated data demonstrate that LC-Opt correctly identifies the most important covariates

To evaluate the ability of LC-Opt for identifying informative combinations of variables, we perform a simulation study. Four models are used to simulate different association between the nutritional variables (the independent variables) and the gene expression. Details of the models are described in the simulation study paragraph in the “Methods” section. For each model we vary the number of independent nutrient variables (*p* − 1) over 9 and 19, with a 10th and 20th variable, respectively, which depends on the first two independent nutrient variables. The sample size *n* varies over 20, 50 and 100. The parameter *r*, which is used to model the level of noise, varies over 0.05, 0.1 and 0.2. We generate repeatedly (100 times) data for each of the models and for each combination of *p* and *n* and calculate LC-Stat for every combination of covariates. To summarize our empirical results, we group each combination of nutrient information according to how many of the informative variables it contain. For example, in Model 1 when *p* = 10, the informative combination is $(X_1,\ X_{10})$. We divide the combinations into three groups $A_1,A_2$ and $A_3$, say with $A_1=\{\text {combinations include all informative variables}\}=\{(X_1,X_{10})\}$, $A_2=\{\text {combinations include only one informative variable}\}=\{(X_1,X_2),\ldots ,(X_1,X_9),(X_2,X_{10}),\ldots ,(X_9,X_{10})\}$ and $A_3 = \{\text {combinations do not include any informative variable}\}$. The box plots of the LC-Stat values for Model 1 and Model 3 (*k* = 3) with respect to each group are shown in Fig. 3 and other settings are shown in Additional file 1: Figure S1.

From Fig. 3 and Additional file 1: Figure S1, we can see that in all cases, where combinations include all informative variables, the LC-Stat is smallest compared to the other combinations. The more informative variables are included in the combination, the smaller the LC-Stat value. As expected, the larger the sample size *n* the smaller LC-Stat (for all cases). When the number of samples decreases, the differences between groups with and without informative variables become smaller. Comparing the results with different number of variables *p*, i.e. *p* = 10 against *p* = 20, we can see that the performance does not deteriorate as *p* becomes larger when *n* is held fixed. Results in these simulations show that our proposed LC-Opt has the ability to identify combinations of variables that have a true non-linear relationship with the response and it is quite stable when the number of variables is approximately as many as the number of observations.

### LC-test: significance testing of the relationship between combination of variables and given gene expression

The LC-Test tests if the selected variables have a non-random relationship with the response. In addition to finding the optimal nutrient components with smallest LC statistics for a response, in practice, we may want to investigate the relationship of a specific response with certain nutrition components of interest. However, in some of the cases, the relationship of the nutrition variables and the genes is not significant, such as in [15], where the GNF of FGF21 with Carbohydrate and Fat is not significant.

We apply our LC-Test method to simulated data to evaluate if the relationship between combination of variables and given a particular gene expression is significant. In this simulation, the data are generated using three models (see “Methods”). Three tests are considered: our proposed LC-Test and two existing F-tests; first, the F-test in the linear regression model (F-test1) which tests under the null hypothesis whether all the regression parameters are equal to zero; and second, the F-test in the linear regression model which additionally considers the interaction term (F-test2) as in [14]. For each model, we perform candidate tests on the informative variables (null scenario) and on randomly selected variables (alternative scenario). We simulate 200 data sets and then calculate the true positive rates (TPRs) and false negative rates (FPRs) at the 5% level and we vary the sample size again over 20, 50 and 100. The results of the top 20 nutrient combinations are shown in Table 1 and the full results can be found in Additional file 1: Table S1.

**Table 1:** Simulation result for LC-Test for identifying informative covariates

	Model 1	Model 2	Model 3
*n* = 100			
LC-Test	0.97 (0.08)	0.99 (0.15)	1.00 (0.22)
F-test1	0.19 (0.04)	0.19 (0.06)	0.69 (0.05)
F-test2	0.17 (0.07)	0.22 (0.03)	0.83 (0.15)
*n* = 50			
LC-Test	0.68 (0.07)	0.87 (0.14)	0.98 (0.18)
F-test1	0.06 (0.06)	0.20 (0.07)	0.32 (0.03)
F-test2	0.08 (0.03)	0.16 (0.04)	0.45 (0.09)
*n* = 20			
LC-Test	0.53 (0.06)	0.69 (0.08)	0.84 (0.22)
F-test1	0.01 (0.03)	0.08 (0.04)	0.15 (0.09)
F-test2	0.01 (0.03)	0.09 (0.05)	0.34 (0.14)

Results for TPR(FPR) at 5% level

From Table 1, it can be seen that in all cases, LC-Test always outperforms the two F-tests in terms of the TPRs while having relative small FPRs. Since the simulated response vector is generated using a non-linear function with respect to the covariates, which violates the assumption of both linear regression models, with and without interaction term; F-test1 and F-test2 result in relatively small TPRs in most cases except in Model 3 when *n* = 100. In the alternative scenario, F-test1 and F-test2 always show slightly smaller FPRs than LC-test, which means that they show similar performance in excluding non-informative variables. Comparing the results across different sample sizes, as expected for all the tests, the TPRs and FPRs somewhat decrease as the sample size *n* is smaller, demonstrating that a larger sample size is preferred. Results in these simulations show that our proposed LC-Test has the ability to identify the combinations of informative covariates, for different models considered.

### Application of LC-N2G on mouse nutrition study reveals nonlinear relationships of dietary components and hub genes

We apply our proposed LC-N2G on a recent mouse nutrition study (see “Methods”). We first use the LC-N2G approach to examine a specific gene ‘FGF21’ to validate the findings presented by Solon-Biet et al. [15].

**Table 2:** The top 20 combinations of nutrition variables selected by LC-Opt for mouse nutrition study

Combination			
Variable 1	Variable 2	LC-Stat	*p* value
Protein	Carbohydrate	85.02	0
Cellulose	Carbohydrate eaten	86.30	0
Carbohydrate	SFA	86.59	0
Carbohydrate	Protein eaten	88.58	0
Carbohydrate eaten	SFA	90.37	0
Carbohydrate	Dry weight food eaten	90.48	0
Dry weight food eaten	Carbohydrate eaten	91.20	0
Cellulose	Protein eaten	92.30	0
Carbohydrate	Cellulose	93.40	0
Protein	Carbohydrate eaten	96.38	0
Protein eaten	Carbohydrate eaten	96.55	0
Cellulose intake	Carbohydrate eaten	98.66	0
Carbohydrate	Energy intake	100.78	0
Carbohydrate	Fat	101.28	0
Protein	Cellulose	102.73	0
Protein eaten	Energy intake	103.63	0.005
Carbohydrate	Cellulose intake	103.69	0
Carbohydrate eaten	Energy intake	104.93	0
Dry weight food eaten	Protein eaten	106.61	0
Fat	Carbohydrate eaten	107.07	0.01

The *p* values are the permutation *p* values from the LC-Test

The results are summarized in Table 2 and Fig. 4a. In Table 2 we see that four sets of variable combinations, where in each either one variable is ‘Protein’ or ‘Protein eaten’ and another is ‘Carbohydrate’ or ‘Carbohydrate eaten’, rank highly, i.e. with small LC statistic value and small corresponding *p* value. This indicates that FGF21 shows a non-linear relationship with protein and carbohydrate. Figure 4a shows the gene expression values for FGF21 with ‘Protein eaten’ and ‘Carbohydrate eaten’, this result confirms prior knowledge that FGF21 (the response variable) is largest in the low protein and high carbohydrate region.

To further investigate the relationship of nutrition and liver gene expression in this dataset, we perform LC-N2G for selected genes of interest to find potential relationships with nutrition variables. This analysis involves five nutrition variables, resulting in $$\left( {\begin{array}{c}5\\ 2\end{array}}\right) =10$$ pairs of combinations and four genes (Ggcx, Slc27a5, Clec4d and Adgrg2). These four hub genes represent four distinct expression profiles selected after applying LC-test and the weighted gene co-expression network analysis (WGCNA) [17] to the gene expression data (see “Methods” for more details).

Figure 4b, c identify the two key nutrition variables that are most associated with genes Slc27a5 and Clec4d, these variable-pairs are (fat, carbohydrate eaten) and (fat, cellulose intake), respectively. In particular, ‘Fat’ is identified to relate to both genes, which is consistent with current studies [18–21] that recognize that fat level affects the expression level of these genes. It is interesting to note that the Spearman correlation of ‘Fat eaten’ with Clec4d and Slc27a5 is − 0.44 and 0.12, respectively. This indicates that LC-N2G has ability to identify nutrition variables that may not have a marginal effect with a considered gene. Further results for gene Ggcx and Adgrg2 can be found in Additional file 1: Figure S2.

## Discussion

We present a novel statistical framework, LC-N2G, that facilitates examination of the relationship between a large number of nutrition variables and gene expression data. This involves developing approaches to estimate the stability of values in the two dimensional surface using a novel local consistency metric. By applying LC-N2G to simulated data, we demonstrate that our method can accurately recover the correct combination of nutrition variables related to a gene of interest. Furthermore, application to a real dataset not only confirms the finding that the response variable FGF21 is largest in the low protein and high carbohydrate region but we also find some potential non-linear relationship between nutrition variables with gene expressions.

We point out that the same form of ${\varvec{W}}({\varvec{M}})$ in Eq. (1) is widely used in machine learning, especially in metric learning, such as in Neighbourhood Component Analysis (NCA) [22]. However, we do not constrain ${\varvec{W}}({\varvec{M}})$ to be normalized as a probability distribution, where the sum of each row equals 1, due to the effect of each sample not being the same when the response is involved. Another interesting fact is that the formula of the LC-Stat is similar to the objective function in spectral clustering [23], which aims at finding a best cut for a given graph structure. Spectral clustering does not make strong assumptions on the statistics of the data and shows good performance when data show some kind of sparsity [24]. Therefore, our method also enjoys these properties. Differences between our method and spectral clustering include that we substitute the square term with the absolute value; this alleviates the influence of possible outliers in the response. The main difference is that in spectral clustering, ${\varvec{M}}$ is pre-specified from certain structure in data, such as a network structure, and then the function optimized over ${\varvec{G}}$, while in our method, the response ${\varvec{G}}$ is observed from data, usually a specific gene expression level, and we try to find a good metric ${\varvec{M}}$, under which LC is small enough.

In LC-Opt, the matrix ${\varvec{M}}$ is a diagonal matrix consisting of 1’s and 0’s on the diagonal only. We use this set because LC-Opt aims to find a combination of nutrition variables with smallest LC statistic. More generally, the matrix ${\varvec{M}}$ could be as general as satisfying being a semi-definite matrix. This makes the LC-Opt flexible enough to find several latent variables, linear combinations of nutrition variables as in principal component analysis (PCA) or partial least squares (PLS) [25], with small LC statistic.

In practice, covariates always have different scales. Most of the macronutrition variables such as protein, fat and carbohydrate are measured in mg, while some micronutrition covariates such as vitamin are measured in International Unit (IU). Different scale imposes non-uniform weight on the $LC({\varvec{M}})$ statistic and the optimization result will tend to select the variable with largest range, that variable which makes the similarity matrix smallest. Thus, we standardized nutrition variables via a *z*-score transformation, that is centering by the mean and scaling by the standard deviation of a variable, to make their scale uniform.

A clear application of the LC-N2G outside of the experimental context in which it has been applied here is in nutritional epidemiology. Increasingly, a range of ‘omics’ data are becoming available in large scale epidemiological cohorts. However, nutritional epidemiologists have struggled to settle on an analytical framework that will move them beyond the ‘single nutrient at a time’ approach that has largely prevailed to date [10]. LC-N2G will allow epidemiologists to reduce the large number of nutritional (and even non-nutritional) variables available to them to those few that are most relevant.

The proposed LC-N2G framework is based on nutrigenomics. However, it is not restricted to exploring the relationship between nutrition and gene expression. Our method has potential to be used in other circumstances to identify a combination of variables that have non-linear relationship with a certain response. One possible application for LC-N2G is in multi-omics, where the data interact in a complex way. The framework of LC-N2G can be easily extended by replacing nutrition data with one of the -omics data such as metabolomics data and using another -omics data or some phenotype as the response, which provides a method for understanding the interaction across multi-platform data.

Finally, we have developed a shiny webpage http://shiny.maths.usyd.edu.au/LC-N2G/ that performs LC-N2G and provides an approach to further investigate the association between nutrients and gene expression for the mouse nutrition study. A demonstration input file consisting of a nutrition and gene expression matrix can be download in this webpage.

## Conclusion

In this paper, we present LC-N2G, a novel statistical framework for ranking and identifying relationships between nutrition and gene expression information. LC-N2G finds combinations with small LC-Stat and tests these combinations using a permutation test to distinguish effects that are different to those from purely random combinations. We applied LC-N2G to both simulation and real datasets and showed that this framework can accurately select combinations of nutrition variables that relate to a gene of interest. LC-N2G is implemented in the software R and all code used in this paper is freely available from https://github.com/SydneyBioX/LC-Vis our GitHub repository.

## Methods

### Identify informative combination of variables using LC-Stat (LC-Opt)

In Eq. (1), if we do not add any constraint on the diagonal matrix ${\varvec{M}}$ for a given gene, the smallest LC is obtained when all entries in ${\varvec{M}}$ equal 1, i.e. all variables are selected. Hence, we consider the following optimization problem for finding smallest local consistency in *k*-dimensional space for a given gene:

4 $$\begin{aligned} \min \limits _{{\text {tr}} ({\varvec{M}})=k} \quad LC({\varvec{M}};{\varvec{G}}), \end{aligned}$$

where $LC({\varvec{M}};{\varvec{G}})$ is defined in Eq. (1), *k* is the pre-specified number of covariates. All examples with visualization in this article consider *k* = 2 only.

The optimization in Eq. (4) is a binary optimization and many methods exist to solve such a problem. In our implementation, we use exhaustive search to calculate the LC-Stat for each considered combination of variables. This is computationally plausible when the number of combinations $$\left( {\begin{array}{c}p\\ k\end{array}}\right)$$ is not large. In situations where $$\left( {\begin{array}{c}p\\ k\end{array}}\right)$$ is large, we could instead use the Genetic Algorithm (GA) [26]. In the implementation of GA, $LC({\varvec{M}};{\varvec{G}})$ is set to be the fitness function. In order to meet the constraints in Eq. (4), a penalty term proportional to $\vert {\text {tr}}({\varvec{M}})-k\vert$ is added to the fitness function.

### Evaluate significance of relationship between combination of variables and given gene expression using LC statistic (LC-test)

In Eq. (1), we can see that if there is a relationship between the gene expression and selected nutrition variables, the LC-Stat should be small. For given nutrition variables $X_1,\ldots ,X_k$ selected by the indicator matrix ${\varvec{M}}$ and a gene expression ${\varvec{G}}$, we propose a LC-Test, a permutation test that uses the LC statistic to evaluate if the nutrition-gene relationship is significant. Under the alternative hypothesis of LC-Test, the response is modeled by $G=f(X_1,\ldots ,X_k)+\epsilon ,\epsilon \sim N(0,s),s>0$ and *f* is a non-constant smooth function, *e* is a random noise.

The procedure of LC-test is described as follows: we first calculate $LC({\varvec{M}};{\varvec{G}})$, the observed LC-Stat. Then we randomly permute the gene expression *B* times, and we denote the *b*th permutation as ${\varvec{G}}_b$, $(b=1,\ldots ,B)$, and calculate corresponding $LC({\varvec{M}};G_b$) statistic. The permutation *p* value is then

5 $$\begin{aligned} p_{\text {perm}} = \frac{1}{B}\sum _{b=1}^B {\mathbf {1}}\{LC({\varvec{M}};{\varvec{G}}_b) < LC({\varvec{M}};{\varvec{G}})\}, \end{aligned}$$

where ${\mathbf {1}}(\cdot )$ denotes the indicator function.

### Simulation study

To evaluate the performance of LC-N2G, we consider three data generating models for the (*p* + 1) nutrition variables and response, respectively:

$$\begin{aligned} & X_1,\ldots X_p \sim \,N(0,1),\ X_{p+1}=\frac{|X_1|}{|X_1|+|X_2|}+r\epsilon , \\ & Model \ 1:\ G = \exp \{-(X_1^2+X_{p+1}^2)\}+r\epsilon , \\ & Model \ 2:\ G = \sin \{(\pi /2)(X_1^2+X_{p+1}^2) \}+r\epsilon , \\ & Model \ 3:\ G = \exp \{-(X_1^2+|X_3|)/5\}+\exp \{-X_{p+1}^2/5 \}+r\epsilon , \\ & Model \ 4:\ G = \exp \{-(X_1^2+X_2^2)/2\}+\exp \{-(X_3^2 + X_{p+1}^2)/2 \}+r\epsilon , \end{aligned}$$

where $\epsilon \sim N(0,1)$ is random noise.

In these three models, we set the sample size *n* equal to 20, 50, 100 and the number of independent and identically distributed variables (*p* − 1) equals to 9 and 19, respectively, generating a *p*th covariate being a non-linear function of $X_1$ and $X_2$. For this non-linear transformation, we choose a composition fraction as is common in nutrigenomics [9, 15, 16], e.g. a protein–carbohydrate proportion. Parameter *r* is used to model the level of noise and we vary *r* over 0.05, 0.1 and 0.2.

In Model 1, the response density has a single peak, as occurring regularly in real data (see e.g in [15, 16, 27, 28]). Many gene expression and other kind of physiological variables with respect to the protein intake and carbohydrate intake have this kind of distribution. In Model 2, we try to consider a more complex kind of response, that is with more than one peak with respect to the informative variables. In Model 3, more than 2 covariates are involved in explaining *G*, we use both *k* = 2 and *k* = 3 to explore the results of LC-Opt for combination identification with different setting of *k*.

### Mouse nutrition study

#### Data description

We apply our LC-N2G on a recent mouse nutrition study, which has been investigated in Solon-Biet [15]. The dataset consist of 176 mice that were fed with different type of diet and water. Diets varied in protein (P), carbohydrate (C), fat (F), and energy (E) content. Energy manipulations were done through the addition of cellulose, allowing for low, medium, and high energy density diets (8, 13, and 17 kJ/g). Spearman correlation between nutrition variables are shown in Additional file 1: Figure S3.

The liver microarray gene expression studies were performed on 48 livers across all diets with a total of 21,800 genes measured. Gene expression was normalised using the rma method [29]. Here, we consider 11 nutrients and all the genes. After excluding the samples with missing values, 42 samples remain for further consideration. In previous work [15], gene FGF21 was investigated and has been found to be maximally elevated under low protein, high carbohydrate intakes via NGF.

#### Selection of nutrition variables and genes of interest

To investigate the relationship of gene expression and nutrition variables, we select some representative genes in the dataset.

For simplicity, in this study 6 nutrition variables are excluded to avoid combinations with redundant information, such as (C, C eaten), (F, F eaten) etc. and 5 nutrition variables (P eaten, C eaten, F eaten, Cellulose intake and SFA) are selected. We perform LC-Test on these 5 nutrition variables on each gene. Then genes with no significant associations to any combination of nutrition variables, i.e. LC-Test *p* value larger than 0.05, are removed. This results in 1851 genes (see Additional file 2: Table S2). Finally, genes are clustered into four modules using WGCNA [17] and the corresponding hubs are Ggcx, Slc27a5, Clec4d and Adgrg2. We use these selected genes and nutrition variables to analysis their potential relationship using LC-N2G (see “Results” section).

## Additional files

**Additional file 1: Figure S1**. Simulation results for LC-Opt for identifying combinations for Model 2, Model 3 (k = 2) and Model 4 (k = 4, k = 3). **Figure S2**. LC-N2G results for mouse nutrition study(Gene Ggcx and Adgrg2). **Figure S.**. Spearman correlation between nutrition variables in mouse nutrition study. **Table S1**. All combinations of nutrition variables for mouse nutrition study.

**Additional file 2: Table S2**. Results for LC-Test on all combination of nutrition variables and all genes in mouse nutrition study.

## Data Availability

The accession number for the microarray data reported in this paper is GEO: GSE85998.

## Abbreviations

NGF: Nutritional geometry framework
LC-N2G: Local consistency nutrition to graph
LC: Local consistency
LC-Opt: Local consistency optimization
LC-Test: Local consistency test
TPRs: True positive rates
FPRs: False negative rates
WGCNA: Weighted gene co-expression network analysis
NCA: Neighbourhood component analysis
PCA: Principal component analysis
PLS: Partial least squares
GA: Genetic algorithm
P: Protein
C: Carbohydrate
F: Fat
