# Supplementary material for: LC-N2G: a local consistency approach for nutrigenomics data analysis
Source: BMC Bioinformatics. 2020 Nov 17;21:530. doi: 10.1186/s12859-020-03861-3 (PMC7672905; doi:10.1186/s12859-020-03861-3)
Supplement: Supplementary file 1 — Additional file 1: Figure S1. Simulation results for LC-Opt for identifying combinations for Model 2, Model 3 (k = 2) and Model 4 (k = 4, k = 3). Figure S2. LC-N2G results for mouse nutrition study(Gene Ggcx and Adgrg2). Figure S.. Spearman correlation between nutrition variables in mouse nutrition study. Table S1. All combinations of nutrition variables for mouse nutrition study. [file 12859_2020_3861_MOESM1_ESM.docx]

A Local Consistency Visualization (LC-Vis) Method for Nutrigenomics

Supplementary material

**Xiangnan Xu^1,2^, Samantha M Solon-Biet^2,3^, Alistair Senior^2,3^, David Raubenheimer^2^, Stephen J Simpson^2,3^, Luigi Fontana^2,4^, Samuel Mueller^1*†^and Jean YH Yang^1,2*†^**

1. School of Mathematics and Statistics, The University of Sydney, NSW 2006, Australia
2. Charles Perkins Centre, The University of Sydney, NSW 2006, Australia
3. School of Life and Environmental Sciences, The University of Sydney, NSW 2006, Australia.
4. Sydney Medical School, The University of Sydney, NSW 2006, Australia.


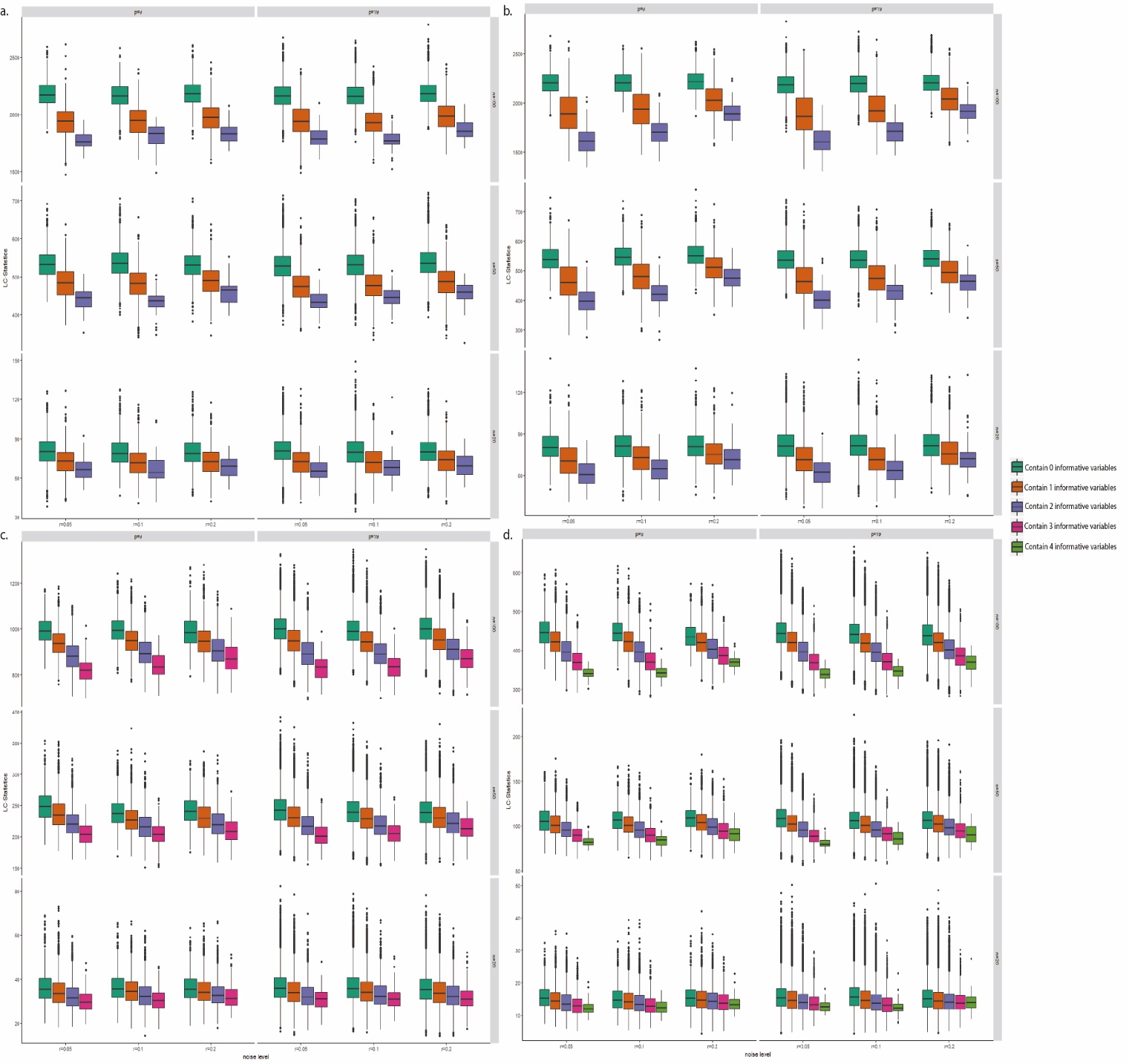


Figure S1 Simulation results for LC optimization for identifying combinations. The combinations are divided into 5 groups according to the informative variables it included. Each model is evaluated under 3 different noise level with different parameter r in the model. a) Boxplots of LC-Statistics for different combination groups of Model 2. b) Boxplots of LC-Statistics for different combination groups of Model 3 with k = 2. c) Boxplots of LC-Statistics for different combination groups of Model 4 with k = 3. d) Boxplots of LC-Statistics for different combination groups of Model 3 with k = 4. In a), b), c) and d) total number of informative variables are 2, 3, 4 and 4 respectively.


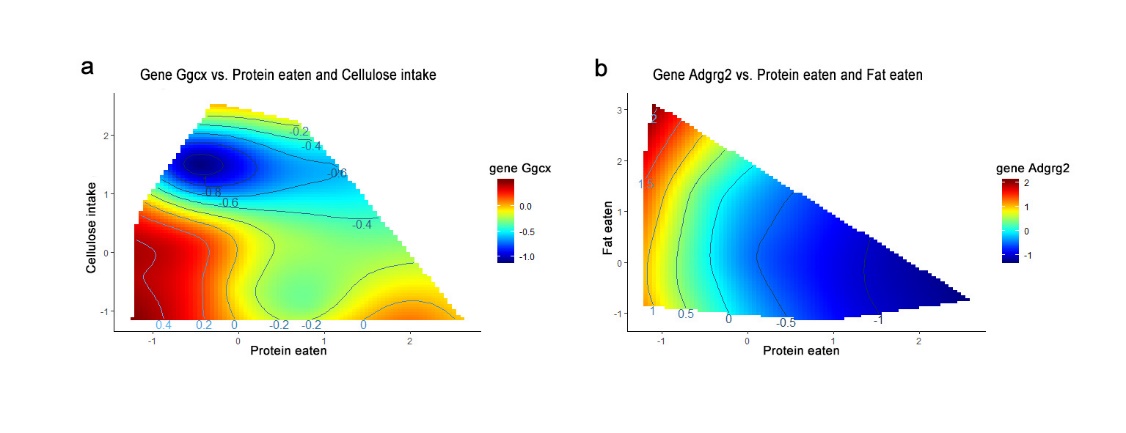


Figure S2 LC-Vis results for mouse nutrition study. a)-b) GFN of Ggcx and Adgrg2 with the informative combination identified by LC-Vis. Ggcx and Adgrg2 are hub genes by WGCNA. Another 2 hub genes Slc27a5 and Clec4d are shown in Figure 4.


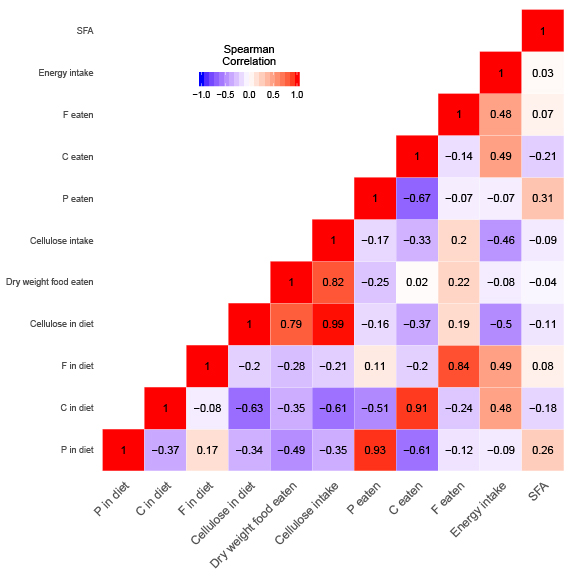


Figure S3 Spearman correlation between nutrition variables in mouse nutrition study

Table S1. All combinations of nutrition variables for mouse nutrition study. The p-values are the permutation p-values from the LC-test.

| Combination | |  |  |
| --- | --- | --- | --- |
| Variable 1 | Variable 2 | LC Statistic | p-value |
| Protein | Carbohydrate | 85.02 | 0 |
| Cellulose | Carbohydrate eaten | 86.30 | 0 |
| Carbohydrate | SFA | 86.59 | 0 |
| Carbohydrate | Protein eaten | 88.58 | 0 |
| Carbohydrate eaten | SFA | 90.37 | 0 |
| Carbohydrate | Dry weight food eaten | 90.48 | 0 |
| Dry weight food eaten | Carbohydrate eaten | 91.20 | 0 |
| Cellulose | Protein eaten | 92.30 | 0 |
| Carbohydrate | Cellulose | 93.40 | 0 |
| Protein | Carbohydrate eaten | 96.38 | 0 |
| Protein eaten | Carbohydrate eaten | 96.55 | 0 |
| Cellulose intake | Carbohydrate eaten | 98.66 | 0 |
| Carbohydrate | Energy intake | 100.78 | 0 |
| Carbohydrate | Fat | 101.28 | 0 |
| Protein | Cellulose | 102.73 | 0 |
| Protein eaten | Energy intake | 103.63 | 0.005 |
| Carbohydrate | Cellulose intake | 103.69 | 0 |
| Carbohydrate eaten | Energy intake | 104.93 | 0 |
| Dry weight food eaten | Protein eaten | 106.61 | 0 |
| Fat | Carbohydrate eaten | 107.07 | 0.01 |
| Cellulose intake | Protein eaten | 108.92 | 0 |
| Carbohydrate | Fat eaten | 110.68 | 0.005 |
| Carbohydrate eaten | Fat eaten | 111.87 | 0.005 |
| Protein eaten | SFA | 117.82 | 0.005 |
| Carbohydrate | Carbohydrate eaten | 119.78 | 0.55 |
| Protein | Cellulose | 120.47 | 0 |
| Fat | Protein eaten | 124.19 | 0.04 |
| Protein | Energy intake | 126.36 | 0.06 |
| Protein | Dry weight food eaten | 126.46 | 0.01 |
| Protein eaten | Fat eaten | 129.14 | 0.005 |
| Protein | SFA | 151.49 | 0.065 |
| Cellulose | SFA | 154.74 | 0.24 |
| Protein | Fat eaten | 159.76 | 0.255 |
| Energy intake | SFA | 163.56 | 0.19 |
| Protein | Fat | 170.38 | 0.43 |
| Cellulose | Energy intake | 175.52 | 0.35 |
| Dry weight food eaten | SFA | 178.76 | 0.395 |
| Cellulose intake | SFA | 181.43 | 0.38 |
| Protein | Protein eaten | 182.53 | 0.705 |
| Dry weight food eaten | Energy intake | 196.99 | 0.83 |
| Fat | Cellulose | 199.72 | 0.915 |
| Fat | SFA | 203.85 | 0.745 |
| Cellulose intake | Energy intake | 204.12 | 0.625 |
| Fat eaten | SFA | 210.48 | 0.725 |
| Fat eaten | Energy intake | 216.28 | 0.405 |
| Cellulose | Fat eaten | 216.32 | 0.955 |
| Fat | Energy intake | 216.62 | 0.425 |
| Fat | Dry weight food eaten | 220.02 | 1 |
| Fat | Cellulose intake | 231.77 | 0.97 |
| Cellulose | Dry weight food eaten | 232.46 | 0.57 |
| Dry weight food eaten | Fat eaten | 237.37 | 1 |
| Cellulose intake | Fat eaten | 253.59 | 0.99 |
| Dry weight food eaten | Cellulose intake | 280.98 | 0.845 |
| Cellulose | Cellulose intake | 300.42 | 0.58 |
| Fat | Fat eaten | 355.06 | 1 |
